# Sensor Data Acquisition and Multimodal Sensor Fusion for Human Activity Recognition Using Deep Learning

**DOI:** 10.3390/s19071716

**Published:** 2019-04-10

**Authors:** Seungeun Chung, Jiyoun Lim, Kyoung Ju Noh, Gague Kim, Hyuntae Jeong

**Affiliations:** SW · Contents Basic Technology Research Group, Electronics and Telecommunications Research Institute, Daejeon 34129, Korea; kusses@etri.re.kr (J.L.); kjnoh@etri.re.kr (K.J.N.); ggkim@etri.re.kr (G.K.); htjeong@etri.re.kr (H.J.)

**Keywords:** mobile sensing, sensor position, human activity recognition, multimodal sensor fusion, classifier-level ensemble, Long Short-Term Memory network, deep learning

## Abstract

In this paper, we perform a systematic study about the on-body sensor positioning and data acquisition details for Human Activity Recognition (HAR) systems. We build a testbed that consists of eight body-worn Inertial Measurement Units (IMU) sensors and an Android mobile device for activity data collection. We develop a Long Short-Term Memory (LSTM) network framework to support training of a deep learning model on human activity data, which is acquired in both real-world and controlled environments. From the experiment results, we identify that activity data with sampling rate as low as 10 Hz from four sensors at both sides of wrists, right ankle, and waist is sufficient in recognizing Activities of Daily Living (ADLs) including eating and driving activity. We adopt a two-level ensemble model to combine class-probabilities of multiple sensor modalities, and demonstrate that a classifier-level sensor fusion technique can improve the classification performance. By analyzing the accuracy of each sensor on different types of activity, we elaborate custom weights for multimodal sensor fusion that reflect the characteristic of individual activities.

## 1. Introduction

As wearable devices are widely used nowadays, Human Activity Recognition (HAR) has become an emerging research area in mobile and wearable computing. Recognizing human activity leads to a deep understanding of individuals’ activity patterns or long-term habits, which contributes to developing a wide range of user-centric applications such as human-computer interaction [1,2], surveillance [3,4], video streaming [5,6], AR/VR [7], and healthcare systems [8,9]. Although activity recognition has been investigated to a great extent in computer vision area [10,11,12], the application is limited to a certain scenario that equips pre-installed cameras with sufficient resolution and guaranteed angle of view. On the other hand, wearable sensor approach allows continuous sensing during daily activities without spatio-temporal limitation [13,14], since devices are ubiquitous and do not require infrastructural support.

Although many commercial fitness trackers or smartwatches adopt wrist-mountable form due to the ease of accessibility, no consensus has been made regarding the positioning of sensors and their data acquisition details. For instance, there are many public datasets available which collect data from Inertial Measurement Units (IMU) attached on different parts of the human body such as ear, chest, arm, wrist, waist, knee, and ankle with various parametric settings [15,16,17,18]. Data acquisition details may vary depending on the type of activities investigated in the research. For example, a sensor on waist with low sampling rate may be sufficient for recognizing simple activities (i.e., coarse granularity) such as walking and sitting. To detect combinatorial activities with finer granularity including eating and driving, on the other hand, a single sensor attached on the waist may not give a satisfactory performance. In this work, we focus on Activities of Daily Living (ADLs) including eating and driving activity, and limit the scope of recognition to basic human activities that constitute ADLs. We expect to obtain a more explainable and structural understanding of daily routines or personal lifestyles through the analysis on the primary unit of activity.

Previous works in HAR literature rely on predictive modeling approaches used in statistics and stochastic process [19,20] (e.g., decision tree, kNN, SVM). Therefore, it requires an expert-level understanding of human activities in terms of medical and social science for data analysis and feature extraction that reflects the characteristic of activity dataset. In this paper, we adopt a deep neural network architecture to recognize human activity from raw data input, which has shown promising results when adopted for interpreting human activities without complex data pre-processing for feature extraction [21,22,23].

This paper presents a preliminary study of investigating optimal position of sensors and their data acquisition details [24] by adopting deep learning algorithm for HAR. Our goal is to find the optimal position and combinations of on-body sensors with effective data sampling frequency that cause minimal burden on data collection procedure. The activity data is collected in both real-world and controlled environments through our testbed system that includes eight IMU sensors attached on different parts of the human body. Then, it is trained and examined using a Long Short-Term Memory (LSTM) [25] neural network classifier.

We apply a classifier-level sensor fusion technique on multimodal sensor data by taking the gyroscope and magnetometer into account in addition to the accelerometer data. After training data from each sensor type on the LSTM network, prediction results from base-learner are combined to generate a stacked meta-feature. Leveraging a Random Forest decision tree as an aggregator model, the meta-learner performs training on the meta-features. Finally, this two-level stacking and voting ensemble model makes prediction on the new multimodal sensor data. To reflect different characteristics in detecting ADLs using multiple modalities, we analyze the recognition accuracy of each sensor modality on different types of activity and compute customized weight that fits best for the individual activity.

Key contributions of this research are:

- Build a testbed system and collect activity data in both real-world and lab environments that contain high variations in the signal patterns.
- Analyze activity data to empirically study the data acquisition details regarding the sensor position and parametric settings, and investigate the characteristic of each sensor modality on detecting different types of ADLs.
- Perform sensor fusion on multimodal sensor data by implementing a two-level ensemble model that combines single-modal results from the deep learning activity classifier.

The rest of this paper is organized as follows. Section 2 summarizes previous work closely related to activity recognition and sensor fusion research. Section 3 describes our testbed and experiment protocols used in the study. Section 4 discusses details of our deep learning framework and sensor fusion model developed for data analysis, and Section 5 presents the evaluation results and findings. Section 6 continues the discussion on current limitations and future work. Finally, Section 7 states the final remark.

## 2. Related Work

Human activity recognition has been investigated using various sensor modalities [26]. Besides the body-worn sensors, object sensors such as Radio Frequency Identifier (RFID) tags are widely used to infer human activities based on the movements detected in smart home environments [27]. In addition, ambient sensors that capture the change of environments including light, sound, temperature, pressure, and humidity are combined to detect activity events [28]. This section introduces literature related to on-body sensor-based HAR, especially the one investigating the placement of sensors, mobile sensing and public datasets, multimodal HAR and their sensor fusion techniques.

### 2.1. Sensor Positioning

Selecting optimal placement of sensors on different body positions has been widely studied for various purposes of activity recognition that range from gait monitoring or fall detection for elderly subjects to daily living activities of healthy subjects usually with no impairments [29]. By assuming a single IMU on-body sensor, authors of [30] defined the cross-location activity recognition problem and proposed a transfer learning based HAR model, which adapts a new sensor position based on known sensor placements. We limit the scope of study to ADLs and focus on finding feasible sensor location utilizing minimum number of body-worn sensors.

Authors of [19] classify activities into four broad levels of activity groups such as very low level, low level, medium level, high level (e.g., from static to dynamic level) based on the compendium of physical activities that leverages the rate of energy expenditure. Seven accelerometer sensors are placed on wrist, arm, waist, chest, ankle, and knee in addition to the ear-worn sensor. According to the experiment result, knee sensor shows the best performance in recognizing high-level activity (e.g., running), while the next best positions were ear and arm sensors. However, the authors claim that feasibility should be taken into consideration because chest or knee-worn sensors would interfere with daily activities. Therefore, consistent yet realistic position of sensor is recommended, depending on the target application.

Similarly, another work [20] investigated seven activities including walking, walking upstairs, walking downstairs, running, standing, sitting, and lying using data acquired from six sensors placed on wrist, ankle, thigh, chest, lower back, and hip. Experiment results show that the hip was the best single location for activity recognition using SVM, and combining two sensors from different locations can achieve a significant increase in the recognition accuracy. The authors stated that combinations of sensors worn on the upper half and another on the lower half of the body can cover simple daily activities. However, the paper leaves identification of finer-grained activities (e.g., sitting and sitting working at a computer) as a future work.

### 2.2. Mobile Sensing and Public Datasets

Many efforts have been made to collect mobile sensing data for HAR, and there are many datasets publicly available. The ExtraSensory dataset [15,16] aims to analyze behavioral context in-the-wild from mobile sensors. It uses sensors from everyday devices including smartphone and smartwatch, where users were engaged in regular natural behavior and self-reported their context with 100 labels. Among many sensors, motion-reactive accelerometer data was sampled with high frequency: 40 Hz from a smartphone and 25 Hz from a smartwatch for 20 s every one minute. However, as the dataset adopts heterogeneous sensors on limited position of the human body, it is not sufficient to evaluate the recognition performance according to the sensor position and sampling rate.

The UCI smartphone dataset [17] continuously records IMU sensor data from a waist-mounted smartphone with 50 Hz sampling rate. In a lab environment, test subjects performed six activities including walking, walking upstairs and walking downstairs, sitting, standing, and lying. Likewise, the UniMiB-SHAR dataset (University of Milano Bicocca Smartphone-based HAR) [18] takes accelerometer samples at a maximum frequency of 50 Hz from a smartphone located in the front trouser pockets of the subjects. Both UCI and UniMiB-SHAR datasets leverage only one sensor mounted on a single point of the body, which limits the scope of experimental scenario.

Adopting high sampling rate may provide sufficient information for data analysis, but imposes burden on the system with large data size and computation load. On the other hand, applying low sampling rate may fail to capture intrinsic attributes of each activity, which can differentiate one from another. To the best of our knowledge, there has been no consensus made regarding the sampling frequency of motion-reactive sensors, and previous work accepts rates commonly used in literature for activity recognition from data acquired through smartphones. Therefore, we aim to investigate the optimal sampling frequency of body-worn sensors attached on different positions of the human body to collect activity data with low burden.

### 2.3. Deep Learning in Ubiquitous Computing

Deep learning model is renowned for its feature learning ability through neural networks, which accepts a large amount of raw data for training and identify unseen data through knowledge transfer methods [31]. As a derivative of Recurrent Neural Network (RNN), LSTM network model is known to perform well on extracting signal patterns in the input feature space. By accepting input data that spans over long sequences, it is specialized in time series problems, especially in time-series prediction domain. The gated architecture of LSTM manipulates the memory states that model the local dependencies on features. HAR is based on the assumption that motion-reactive sensor signals present discriminative patterns corresponding to different activities. As such activities usually last for a certain period of time when they occur, HAR is regarded as a time-series classification problem with temporal dependency, where input data that are close in space may be dependent while distant sequence of samples in time are assumed as independent. Thus, LSTM network model is renowned for its performance on HAR domains [32]. Papers [33,34] also compared deep-learning methods for HAR, and verified that variants of the LSTM architecture lead to flexible and robust results in several sensor-based HAR datasets.

### 2.4. Multimodal Sensor Fusion

Many efforts have been made to combine multimodal sensor data to ensure the classification accuracy by taking the advantage of diverse data sources. Sensor fusion in HAR field is usually divided into two categories, feature-level and classifier-level fusion. Authors of [16] referred to the feature-level fusion as early fusion, which merges feature data extracted from each modality prior to the classification procedure. Using a single feature vector for the training model is straightforward, but feature compatibility issues regarding heterogeneous sampling frequencies and configuration parameters can deteriorate the classifier performance [35]. Contrarily, classifier-level fusion aims to combine the classification results given by the base-learners trained on different sensor modalities. As class-probability predictions are uniform in their expression, classifier-level ensemble learning techniques are widely adopted to integrate multiple machine learning models [34,35,36]. In this paper, we apply two-level stacking and voting ensembles, which fall into the classifier-level sensor fusion technique.

## 3. Testbed and Data Acquisition

This section first describes the system architecture of the proposed body-worn sensor testbed that acquires human activity data. Then, the experimental protocols for data collection are explained in detail.

### 3.1. Testbed System

**Figure 1:**
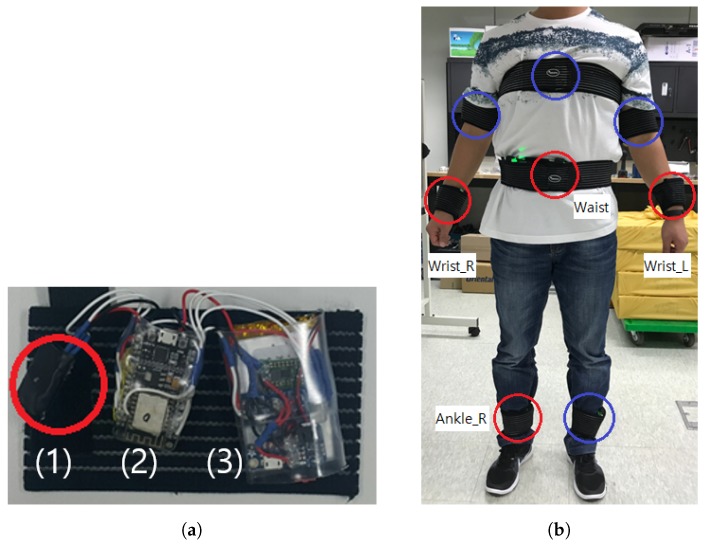
Testbed configuration. (**a**) A body-worn Inertial Measurement Unit (IMU) device consists of (1) a 9-axis IMU sensor (i.e., 3-axis accelerometer, gyroscope, and magnetometer, respectively), (2) a Micro Controller Unit (MCU), and (3) a micro-USB rechargeable Li-Po battery module; (**b**) Among eight body-worn sensors (in circles), only four sensors at left and right wrists, waist, and ankle (in red circles) are adopted for the data analysis.

We build a testbed system that acquires continuous motion data from on-body IMU devices. We adopt a MPU-9250 IMU sensor that samples tri-axial accelerometer, gyroscope, and magnetometer data at maximum 100 Hz frequency. As shown in Figure 1a, each IMU device is implemented on a ESP8266 Micro Controller Unit (MCU) using a Wi-Fi networking and nRF24L01 RF module, which is powered by a 3.7 V–1200 mAh micro-USB rechargeable Li-Po battery. The corresponding battery capacity is empirically verified to guarantee at least 12 h of continuous data collection with maximum sampling frequency. IMU devices are designed to be attached on both wrists, upper arms, ankles in addition to chest and waist of a test subject. Medical Velcro straps are used to fasten devices on the body to reduce signal noise.

**Figure 2:**
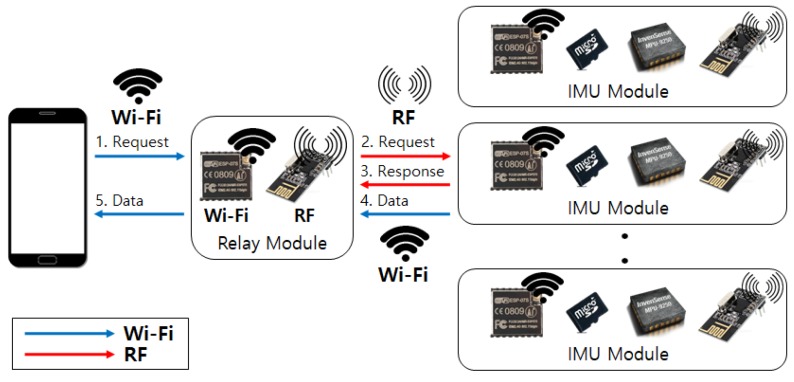
Testbed system consists of a mobile device, a relay module, and multiple IMU sensor devices. (1) Request for soft-AP setting, (2) Radio Frequency (RF) connection request, (3) Response on connection establishment, (4)–(5) Activity data transmission.

We implement a relay module that operates as a data collection hub between IMU devices. As a large amount of activity data is accumulated at each IMU device, we choose Wi-Fi protocol to assure fast and stable data transmission through high bandwidth network channels. Therefore, the relay module first transceives operation commands between IMU devices using the RF protocol to initiate time-synchronized one-to-many communication channels between IMU devices. After establishing a Wi-Fi ad-hoc network through the soft-AP setting, the relay module then can retrieve activity data from each IMU device. When data gathering is completed, the relay module transmits the collated data to an Android mobile device over the Wi-Fi network. Overall communication protocol is illustrated in Figure 2.

### 3.2. ADLs

Statistics Canada annually publishes Time-use survey of the General Social Survey (GSS) [37], which includes respondents’ time spent on a wide variety of day-to-day activities in Canada. In addition, Statistics Korea issues Koreans’ average time-use [38] categorized by mandatory, personal, and leisure time activities. Authors of [18] surveyed ADLs and their occurrence in public HAR datasets, and we find that six primitive activity labels including walking, standing, sitting, lying, walking upstairs, and walking downstairs can cover daily activities listed in time-use survey results.

In addition to the primitive activities, we take three combinatorial activities into account: eating, driving a car, and moving in a car as a passenger. For example, eating activity is an integration of sitting activity (i.e., primitive) and actions using cutlery during a meal (i.e., secondary activity). Similarly, driving a car can be interpreted as a combinations of sitting activity and actions related to handling the steering wheel. In addition, moving in a car as a passenger can include additional actions such as using smartphones while sitting. Table 1 specifies nine activity labels used in our experiments.

### 3.3. Experiment Protocols

As a pilot experiment, we collected activity data from five researchers working at a research institute. Test subjects were composed of two males and three females with ages ranging from 34 to 48 years, heights ranging from 160 cm to 175 cm. All subjects were right-handed with no bodily impairments. Test subjects participated with informed consent and instructed to perform daily activities freely with no restrictions on their body movements. A total of three experiment sessions were carried out on three different workdays, accumulating about three hours of activity data per each subject.

Two different experiment protocols were designed for data collection: a real-world scenario and a controlled environment scenario. Experiments in a real-world scenario were conducted twice during the lunch time of ones’ daily routine. No instructions were given to the test subject with regard to the experiment protocol, so test subjects behaved as usual in a natural manner. An instructor monitored the whole experiment session and manually tagged the corresponding activity labels. The sequential activities labeled during a round trip between the workplace and a restaurant is summarized in Table 2. On the other hand, an instructor informed each target activity and test subjects followed the instruction during the controlled environment scenario. One iteration of experiment is performed to collect activity labels presented in Table 3.

### 3.4. Final Testbed Configuration

At the initial stage of the system design, we developed eight body-worn sensors as shown in Figure 1b. During the primitive study, we observed that applying an excessive number of sensors interrupts identifying the unique characteristic in each activity data. In addition, sensors attached on both ankles did not show significant difference in distinguishing the activities. Therefore, we adopted the sensor on right ankle, and for the same reason, we excluded sensors on upper arms. Participants experienced significant discomfort incurred by the sensor on the chest; we dropped the chest sensor in the final testbed configuration for its feasibility.

## 4. Data Analysis

We extract activity data and their corresponding labels, and segment them using a fixed-width sliding window with 50% overlap. Sliding window size is a base unit to be applied to the data analysis, which can be interpreted as the resolution of activity. By leveraging a long window size, we can handle a stream of activity data as one phase to observe long-term activity patterns or trends, but may fail to extract distinguishing characteristics of each activity if discrete activities are contained in one segment of activity data. On the other hand, a short window size may be suitable when targeting finer primitive actions that occur in a very short period of time, especially with regard to low-level hand gestures such as reach, grasp, and release.

**Figure 3:**
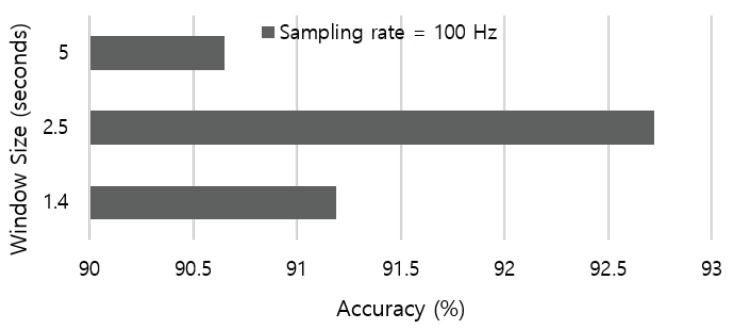
Recognition accuracy according to various length of sliding window.

We aim to perceive coarser-grained activities related to modes of locomotion, which usually lasts for several seconds. Previous work in the HAR literature empirically adopted 5.12 s-long window size [35,39]. Based on the average free-living cadence (i.e., 76 ± 6 steps per minute [40]), we leverage the duration of one gait cycle that ranges from 1.46 to 1.71 s as a baseline. Therefore, we evaluate the recognition accuracy by applying a various sliding window that starts from 1.4 to 5 s. The result in Figure 3 indicates that the 2.5 s-long sliding window outperforms other parameter settings in our dataset.

Deep learning classification models are capable to give better inference results using raw sensor data than the feature data, where pre-processing transformations usually decrease the classification accuracy [21]. Thus, raw sensor data is directly fed into LSTM cells for offline activity classification with no additional feature engineering [22,23].

### 4.1. Deep Learning Architecture

As a variation of RNN architecture, LSTM [25] network is renowned for its capability to learn long-term dependencies in time-series data by the selective forget-and-update procedure. The gated architecture operates the memory states that model the temporal dependencies. Through a sigmoid layer called forget gate, the LSTM cell decides how much information should be saved based on the time dependency of the previous data. Then, the input gate layer creates new candidate values from the input and updates the old cell state to a new one. These LSTM recurrent layers automatically learn feature representations and model the temporal dynamics between cell activation.

**Figure 4:**
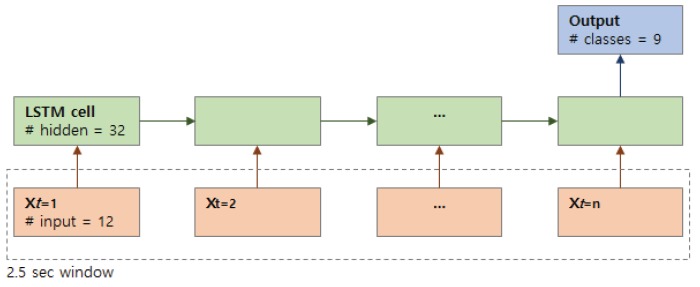
Many-to-one Long Short-Term Memory (LSTM) network architecture used for activity classification with nine classes. *n* stands for the number of samples included in a 2.5 s window.

Our deep learning framework accepts time-series of sensor data as an input vector and convert them to a probability vector at the output for activity classification. A single data vector is assigned per time step, where a pre-defined number of time steps compose a unit (e.g., 2.5 s) of the time series as shown in Figure 4. The input vector consists of 3-axis accelerometer values sampled from four sensors for each cell, where we apply gyroscope and magnetometer readings for sensor fusion. We implemented a LSTM network using Google’s TensorFlow framework by manually tuning the following parameters: batch size = 150, number of stacked LSTM layer = 1, hidden neuron size = 32, number of steps = 25, keep probability = 0.8.

### 4.2. Sensor Fusion

**Figure 5:**
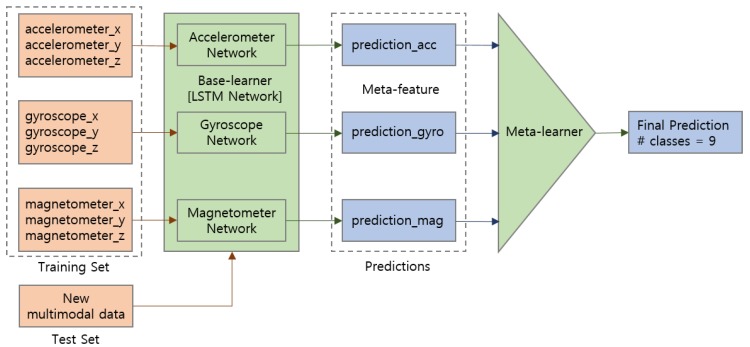
Classifier-level ensemble for multimodal sensor fusion. Prediction results from each sensor modality are used as meta-features for the meta-learner.

In addition to the accelerometer data, we also consider values collected from gyroscope and magnetometer sensors for multimodal sensor fusion to ensure the activity recognition performance. Leveraging the LSTM network as a base learner, we employ classifier-level fusion to combine results from multimodal data as shown in Figure 5. For each sensor modality including accelerometer, gyroscope, and magnetometer, prediction results from LSTM network indicating the classification probabilities of each activity class are accepted as meta-features. As a meta-learner, we first adopt stacking ensemble with 10-fold cross-validation, which trains the aggregator model on the stacked class-probabilities. We also apply simple soft-voting ensemble based on the weighted average of class-probabilities, where the weights are determined according to the prediction accuracy of the base-learner. Finally, the ensemble model makes combined predictions for the new multimodal input data.

## 5. Evaluation

In this section, we first evaluate the activity recognition performance with different configuration parameters applied using the accelerometer data. We empirically investigate the optimal sampling rate of body-worn IMU sensors and their position on the body. Then, we perform evaluation on the sensor fusion technique with multimodal sensor data.

In the classification problem, class imbalance is common because most datasets do not contain exactly equal number of instances in each class, and this phenomenon also applies to human activities during daily lives. F1-score is a commonly used measure in the class imbalanced settings [41]. In the multi-class classification domain, however, micro-averaging F1-score is equivalent to accuracy which measures a ratio of correctly predicted observation to the total instances. Micro-F1 reflects the unbalanced distributions of samples across classes, placing more weights on the dominant classes. On the other hand, macro-averaging F1-score is a harmonic mean of precision and recall measures, and takes both false positives and false negatives into account based on a per-class average [42].

We basically apply 10-fold cross-validation on the data to generalize the result to an independent dataset. By randomly partitioning the dataset into 10 equal-sized subsamples, it leaves a single subsample (i.e., 10% of total activity data) out as test data, while the remaining nine subsamples (i.e., 90% of data) are used as training data. In this way, the system is tested with unseen dataset. We also perform leave-one-subject-out (LOSO) cross-validation for reference by leveraging data augmentation [43,44,45,46], which is widely adopted in the deep learning research that requires large dataset for model training. We apply the jittering method to simulate additive sensor noise using random parameters as described in [47] for label-preserving augmentation. In this way, we handle limited data availability and secure a large amount of training data to increase robustness of our deep learning model. The mean performance of the cross-validation results is used in the remainder of this paper.

### 5.1. Impact of Sampling Rate

**Figure 6:**
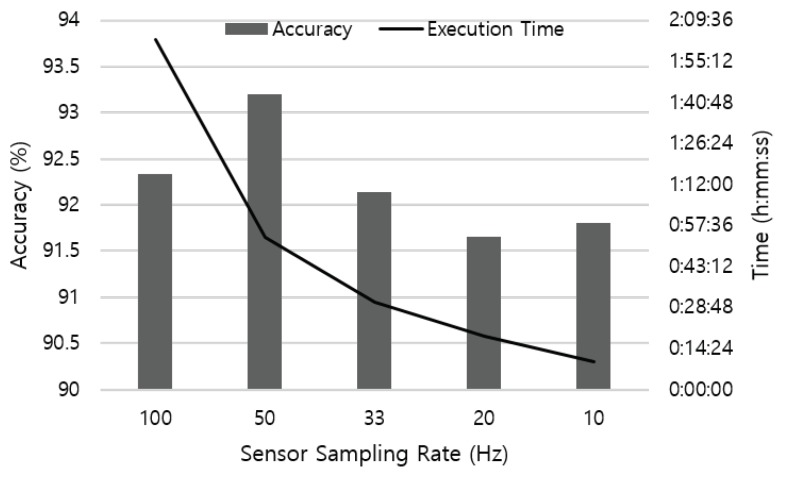
Recognition accuracy and execution time according to different sampling rate of the sensor.

We first tested the recognition accuracy with different sampling rates applied. Starting from 100 Hz, we sub-sampled the sensor data and reduce the frequency to 10 Hz. The number of data was 20,132 when sampling at 100 Hz, and the number slightly varied with different sampling rates applied. Figure 6 shows the test results using four sensors attached on the left and right wrist, right ankle, and waist. As the sampling rate varies, the number of sample entries included in the 2.5 s-long sliding window vary accordingly. From the result, we found that a continuous time-series data with only 25 sensor samples that cover 2.5 s of body movement can successfully recognize the activity without significant loss in the accuracy.

Figure 6 also represents the execution time to perform a 10-fold cross-validation on a desktop computer equipped with Intel Core i7 3.47 Hz CPU. Reduced sampling rate decreases the time to retrieve results from the neural network as the resolution of data becomes coarse. It is an encouraging result for mobile platforms, as devices usually do not have enough resources such as computation power, battery capacity and storage capability. By leveraging lower data sampling frequency, a mobile-based HAR system can operate in a cost-effective manner.

Our findings are in line with the policy of many health trackers in the market that usually support low-frequency sampling rate. For example, a motion and physiological signal tracker Empatica E4 [48] supports the accelerometer data sampling at 32 Hz. In addition, a motion sensor manufacturer [49] provides a calculation tool that estimates maximum data acquisition time depending on the sampling rate of a sensor. According to the calculation, a MetaMotionC IMU sensor can operate up to 11.6 h at 12.5 Hz, while the battery lasts only 5.8 h at 25 Hz sampling frequency, respectively. As HAR applications in-the-wild require continuous and seamless data collection with the day-long battery life, it is worthwhile to identify that low-frequency activity data is still effective in recognizing daily activities.

### 5.2. Impact of Sensor Position and Combinations

**Figure 7:**
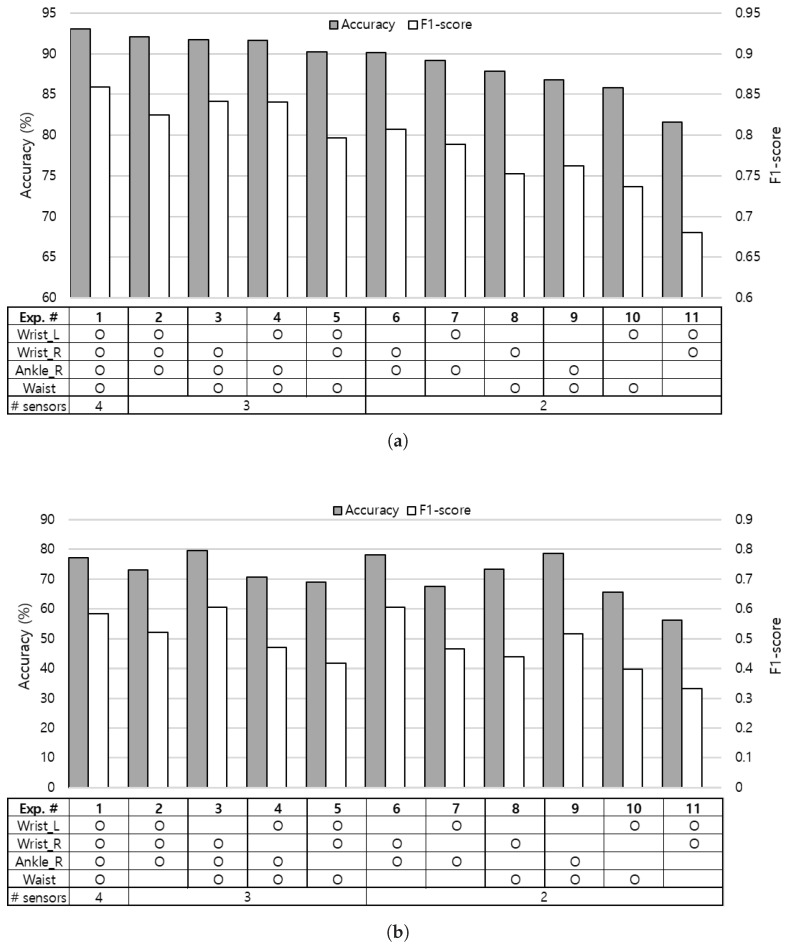
Recognition performance according to different numbers and combinations of body-worn sensors. Both accuracy and F1-score are adopted as the performance measure. (**a**) 10-fold cross-validation result; (**b**) Leave-one-subject-out cross-validation result.

To find an optimal sensor combination for the HAR systems, we investigated the recognition accuracy with different numbers of sensors attached on various locations of the body. The experiment with four sensors on left and right wrists, right ankle, and waist is used as the baseline, which is represented as experiment no.1 in Figure 7. The baseline experiment shows 93.0% of recognition accuracy as shown in Figure 7a. Starting from this baseline, we excluded each sensor in turn and extensively evaluated all sensor combinations on various body positions.

Experiment no.3 that leverages right wrist, right ankle, and waist sensors presents the most competitive results among experiments using three sensors, even when considering inter-subject learning model as shown in Figure 7b. With only two sensors, positioning one on the upper half (e.g., wrists) and the other on the lower half (e.g., ankle or waist) of the body as experiment no.6 performs better than placing both sensors on the upper half (experiment no.11) or the lower half (experiment no.9) of the body. We can expect performance improvement when appending an additional sensor on the lower half of the body to the sensor combinations on the upper half of the body by comparing experiment results of no.2, no.5 to no.11. Similarly, we can observe performance gain by attaching a sensor to the upper half of the body, especially on the right wrist (experiment no.3) to the sensor combinations on the lower half of the body (experiment no.9). From the aspect of the wrist sensor, we found that an additional single sensor on the ankle, waist, and the opposite side of the wrist in sequence can influence the performance when adopting two sensor locations as shown in experiment results [no.6, no.8, no.11] and [no.7, no.10, no.11].

We investigated the contribution of each wrist to the recognition performance by comparing experiments that leverage sensors on each side of the wrist. From the results of experiment [no.3, no.4], [no.6, no.7], and [no.8, no.10] pairs, we observed that the sensor attached on right wrist tends to perform better than the left wrist. This is due to the fact that all five test subjects were right-handed persons, and eating activity is included in the experiment. Next, we explored the impact of sensors on the lower half of the body by comparing results of experiment [no.2, no.5], [no.6, no.8], and [no.7, no.10] pairs, and noticed that the sensor on the right ankle plays a more critical role than the sensor on the waist in the activity recognition.

By taking the trade-off between accuracy and feasibility into account, we concluded that placing sensors on the right wrist and right ankle can give reasonable performance when adopting two sampling positions, and an additional sensor may be placed on the waist to achieve better recognition accuracy.

### 5.3. Impact of Multimodal Sensor Fusion

**Figure 8:**
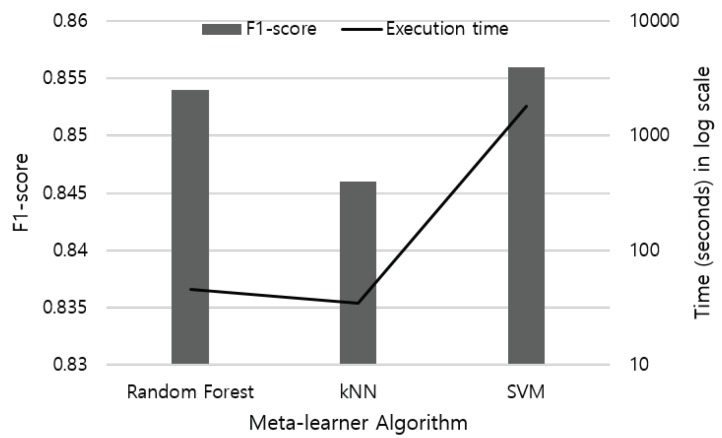
Performance and execution time of meta-learner models used in stacking ensemble.

For multimodal sensor fusion, we adopt stacking ensemble with various combinations of sensor modality. The meta-learner accepts stacked class-probabilities of each sensor modality to train the classification model. As shown in Figure 8, we compare the performance of Random Forest, kNN, and SVM when applied as a meta-learner for stacking ensemble, and adopt Random Forest algorithm. We also apply voting ensemble as the meta-learner that combines the accelerometer, gyroscope, and magnetometer sensor data. In hard-voting, the final class label is determined as the class label that has been predicted most frequently by the classification models. On the other hand, soft-voting predicts the class labels with the highest class probability, averaged over all the individual classifiers. Weights are determined based on the accuracy of each sensor modality, while each sensor may show different performance depending on the type of activity. Therefore, we elaborate weights according to the characteristic of each activity.

**Figure 9:**
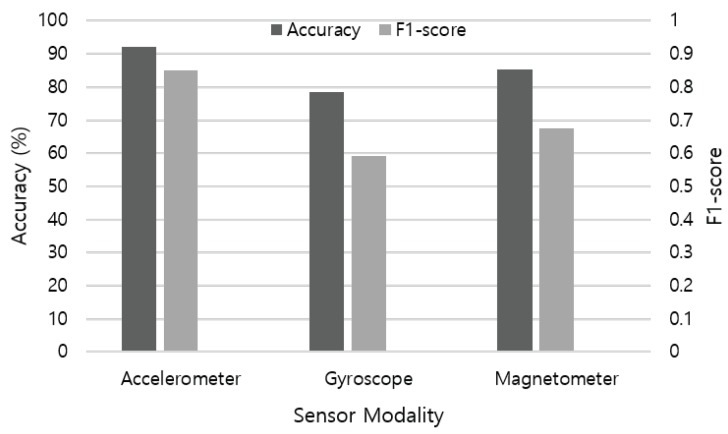
Recognition accuracy and F1-score measure of each sensor modality using the LSTM network.

First, we evaluate the performance of single sensor modality in activity recognition. Figure 9 illustrates the recognition accuracy of each sensor data applied to the LSTM base-learner model. The accelerometer shows the best performance (92.18%) among IMU sensors, followed by magnetometer (85.22%) and gyroscope (78.33%). As each sensor measures different physical aspects, contribution in recognizing ADLs differs according to the characteristic of activities. For example, an accelerometer is useful in measuring changes in velocity and position, and in consequence detecting small movements. By leveraging multiple axes, an accelerometer is also widely used as an absolute orientation sensor in the up-down plane. A gyroscope measures changes in orientation and rotational velocity, but requires calibration from a known orientation to achieve accuracy due to high amount of drift. A magnetometer is useful to determine absolute orientation from magnetic north with low drift over time, but shows poor accuracy for fast movements. Therefore, these multimodal sensors are usually combined to compensate the performance of each other.

**Figure 10:**
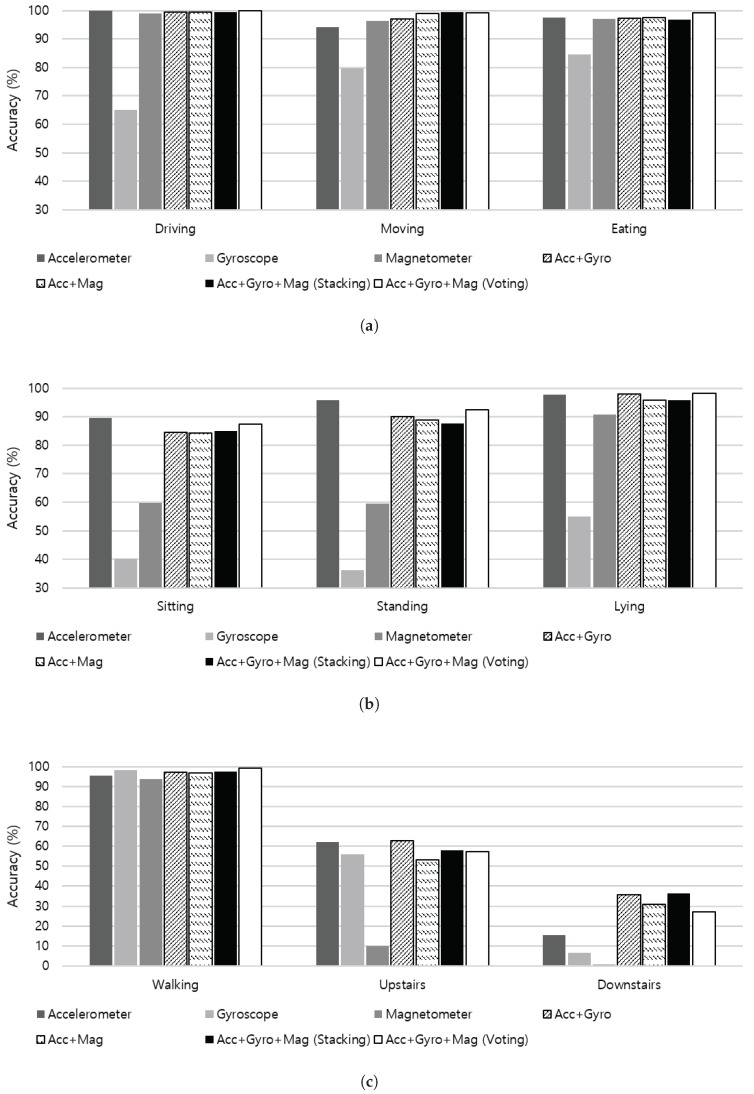
Recognition accuracy of a group of activities using multiple sensor modalities. (**a**) A group of combinatorial activities of sitting, including driving, moving in a car (as a passenger), and eating; (**b**) A group of static activities including sitting, standing, and lying; (**c**) A group of activities that involves walking, including walking upstairs and walking downstairs.

To investigate the contribution of each sensor on recognizing different activities, we evaluate the recognition accuracy of each sensor modality on individual activities as shown in Figure 10. Activities illustrated in Figure 10a are a group of combinatorial activities of sitting, including driving, moving in a car as a passenger, and eating. As a vehicle in operation generates consistent vibration and periodic acceleration, both moving in a car as a passenger and driving activities are well distinguished from sitting activities in different environment. Additionally, eating activity includes repetitive hand movements while sitting, which incurs various yaw, pitch, and roll rotations that can be effectively captured through the gyroscope sensor. Although driving, moving in a car, and eating activities can be considered as semi-static because activities occur during sitting, individual sensor modality shows high recognition accuracy independent from each other due to additional gestures. Both combinations of sensor fusion (i.e., accelerometer + gyroscope and accelerometer + magnetometer) show a similar contribution on the performance improvement.

Figure 10b presents the recognition accuracy of a group of static activities including sitting, standing, and lying. In static position, gravity of the earth may be the major reading of the accelerometer, which accordingly reflects the tilt angle of the body posture in the signal. No remarkable measurements take place at the gyroscope sensor, while the magnetometer measures heading of the sensor. Therefore, the accelerometer shows outstanding performance in detecting different static postures. Furthermore, fusing additional sensors to the accelerometer data even degrades the accuracy in detecting sitting and standing activities.

**Figure 11:**
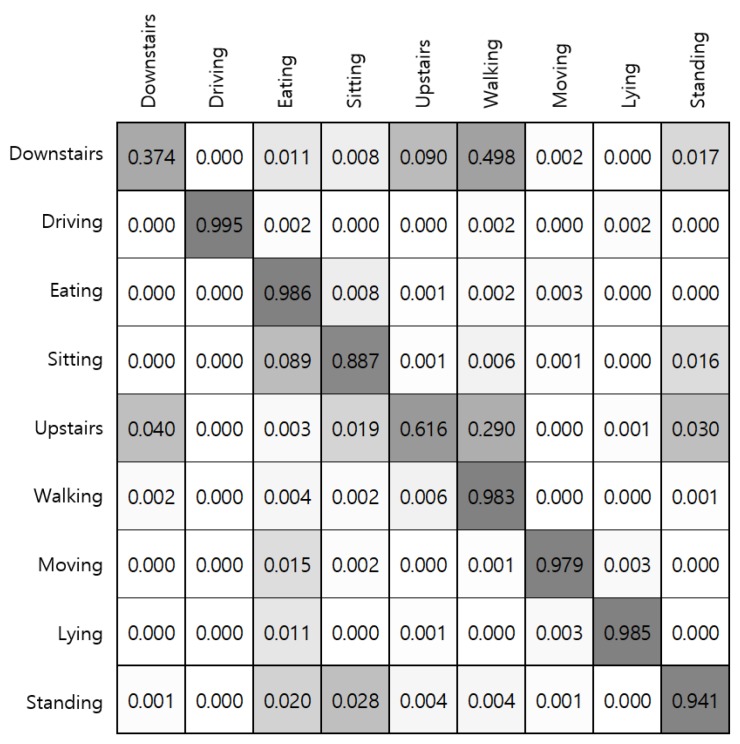
Confusion matrix for each activity. The x-axis stands for the ground truth and the y-axis denotes predicted labels through voting ensemble.

Figure 10c summarizes results of a group of activities that involves walking, including walking upstairs and downstairs. As walking activity involves highly dynamic physical movements, it is detected with high accuracy using each sensor modality, and sensor fusion also contributes to the recognition performance. However, experiments in making distinctions between stair walking and walking on the plain produced equivocal results. As shown in Figure 11, both walking upstairs and walking downstairs are incorrectly predicted as walking with high probabilities, where the x-axis of the confusion matrix stands for the ground truth and the y-axis denotes predicted labels. According to our experiments, fusing the accelerometer and gyroscope sensor data through stacking ensemble led to substantial improvement in detecting stair walking activities.

**Figure 12:**
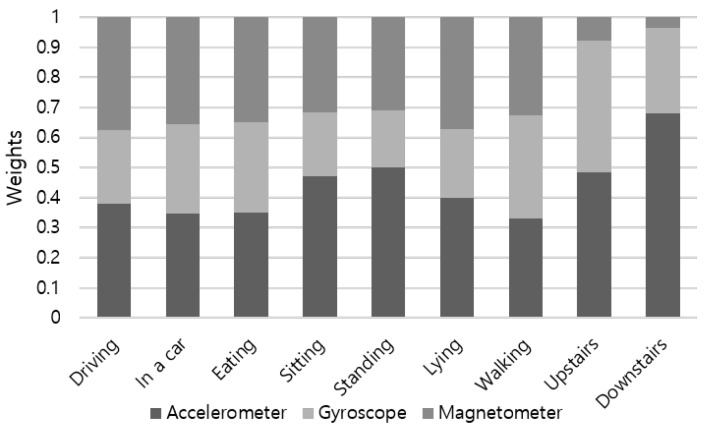
Proportion of weights of each sensor is computed based on the recognition accuracy.

**Figure 13:**
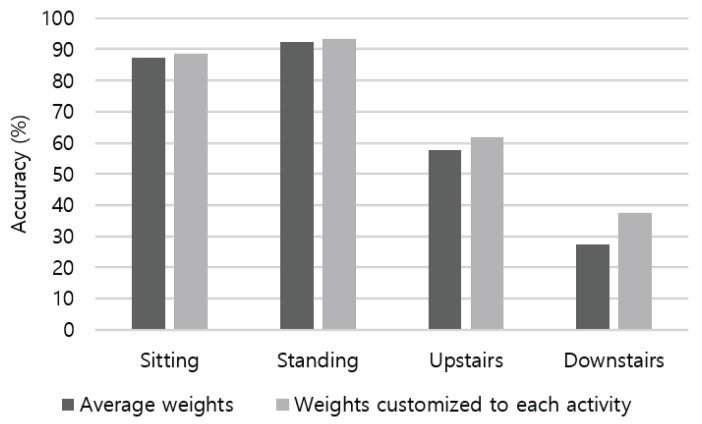
Recognition accuracy increases when applying weights that are customized to each activity.

In soft-voting ensemble, sensor modalities are weighted by their demonstrated accuracy shown in Figure 9, which covers all activity types. Weight proportion of the accelerometer, gyroscope, and magnetometer is applied as 0.36:0.31:0.33 by default. To consider different characteristics of each sensor on detecting various activities, we choose weights by the activity basis. Figure 12 summarizes the proportion of weights computed using the recognition accuracy of sensor modalities. For a group of combinatorial activities of sitting, including driving, moving in a car as a passenger, and eating activities, both the accelerometer and magnetometer had similar weights (e.g., 0.35∼0.38). Among static activities, weight proportion of sitting showed analogous result to standing: 0.49:0.2:0.31 for the accelerometer, gyroscope, and magnetometer, respectively. As weights for walking upstairs and walking downstairs are noticeably different from other activities, applying activity-specific weight attributes substantial increment to the recognition accuracy. As presented in Figure 13, customized weight of each activity contributed to accuracy increase of 7.65% and 36.97% in the case of walking upstairs and downstairs, respectively.

**Figure 14:**
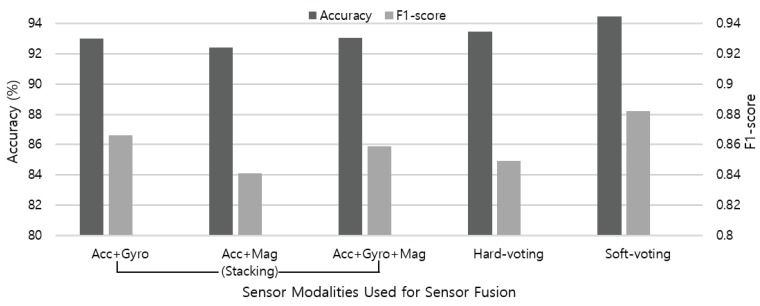
Recognition accuracy and F1-score measure of stacking ensemble using various combinations of sensors, and two voting ensemble techniques adopting all sensor modalities.

Finally, Figure 14 summarizes the sensor fusion results using both stacking and voting ensemble techniques. Overall, the contribution of gyroscope to the recognition accuracy is higher than the magnetometer when combined with the accelerometer data. Although the gyroscope sensor data is used without calibration procedure, the results indicate that applying sensor data from additional modalities can contribute to improving the recognition accuracy of nine activities up to 93.07%. In addition, soft-voting achieves higher overall performance (94.47%) than hard-voting (93.48%), because it can give more weight to highly confident votes. By applying multimodal sensor fusion, we achieved 2.48% of performance improvement compared to the single modal-based activity recognition. Even considering the balance between precision and recall measures, our model performs well and seems to be sufficiently competitive with uneven class distribution.

## 6. Limitations and Future Work

Test subjects: As this work is initially designed to investigate the testbed system before actual distribution on large-scale subjects in real-world environment, experiments took place within the institution on a small number of test subjects. Based on our findings, we plan to adopt off-the-shelf wearable sensors in a lightweight form to improve the practicality of our testbed system. By tuning the systematic parameters, our refined testbed will enable a large-scale and long-term experiment that captures human activity in-the-wild.

Meta-learner: We have evaluated the meta-learner using classical classification algorithms including RF, kNN, and SVM, which are widely adopted in the literature. We plan to implement more classification algorithms with enhanced performance [50], and also extend our meta-learner using deep learning models to improve the recognition accuracy.

Future work: We further expect to gain a deep understanding on individuals’ daily routines or long-term habits through a longitudinal study, which can contribute to the anomaly and pattern detection that may change over time. We also aim to apply additional sensing modalities such as environmental (e.g., background audio recordings, GPS or network locations) and physiological (e.g., electrocardiogram, electro-dermal activity) sensors. For sensor fusion, learning weights of each sensor modality on-line and in a real-time fashion may help to achieve better recognition accuracy and fast adaptation on new experimental settings.

## 7. Conclusions

In this paper, we perform an empirical study about the on-body sensor positioning and data acquisition details for HAR systems. We train the raw activity data collected in both real-world and controlled environments using a deep learning framework, which automatically learns features through neural networks without heuristic domain knowledge applied.

By leveraging the LSTM network that manipulates temporal dependencies on the time-series activity data, we identify that low sampling rate—as low as 10 Hz—is sufficient for the activity recognition. As low sampling frequency reduces the system burden by preserving battery and storage capacity, it consequentially allows prolonged data collection for HAR applications that usually operate on resource-hungry mobile devices. Our experiment result indicates that only two sensors attached on the right wrist and right ankle can present reasonable performance in recognizing ADLs including eating and driving (e.g., either as a driver or a passenger) activity. In other words, placing one sensor on the upper half and the other on the lower half of the body is recommended as the practical parametric setting of sensor position for further HAR research.

Additionally, we investigate the impact of sensor fusion by applying a two-level ensemble technique including stacking and voting on the multimodal sensor data, and demonstrate the performance improvement. By analyzing the recognition accuracy of each sensor on different types of activity, we elaborate custom weights for sensor modalities which can reflect the characteristic of individual activities. 

## Figures and Tables

**Table 1:** Activity labels.

No.	Activity Label	Main Activity	Secondary Activity
1	Walking	Walking	-
2	Walking Upstairs	Walking	Going up
3	Walking Downstairs	Walking	Going down
4	Sitting	Sitting	-
5	Eating	Sitting	Eating and gesture
6	Driving (Driver)	Sitting	Handling the steering wheel
7	Moving (Passenger)	Sitting	On a car
8	Standing	Standing	-
9	Lying	Lying	-

**Table 2:** Experiment protocol in the real-world scenario (two iterations).

No.	Activity	Duration (min)
1	Walking Downstairs	3
2	Walking	5
3	Driving or Moving	5
4	Sitting	5
5	Eating	20
6	Driving or Moving	5
7	Walking	10
8	Sitting	5
9	Walking	20
10	Walking Upstairs	3
**Total**		**81**

**Table 3:** Experiment protocol in the controlled environment (one iteration).

No.	Activity	Duration (min)
1	Standing	5
2	Sitting	5
3	Eating	10
4	Lying	5
5	Walking Downstairs	3
6	Walking Upstairs	3
**Total**		**31**
